# A genetic assay for gene essentiality in *Clostridium*

**DOI:** 10.1016/j.anaerobe.2016.07.007

**Published:** 2016-12

**Authors:** David J.F. Walker, John T. Heap, Klaus Winzer, Nigel P. Minton

**Affiliations:** a Clostridia Research Group, BBSRC/EPSRC Synthetic Biology Research Centre (SBRC), School of Life Sciences, Centre for Biomolecular Sciences, University of Nottingham, University Park, Nottingham, NG7 2RD, United Kingdom; b Nottingham Digestive Disease Centre, NIHR Biomedical Research Unit, The University of Nottingham, University Park, Nottingham, United Kingdom

**Keywords:** *Clostridium difficile*, ClosTron, Merodiploid, Gene essentiality, Therapeutic target

## Abstract

Essential genes of pathogens are potential therapeutic targets, but are difficult to verify. Here, gene essentiality was determined by targeted knockout following engineered gene duplication. Null mutants of candidate essential genes of *Clostridium difficile* were viable only in the presence of a stable second copy of the gene.

## Highlights

• A rapid assay to demonstrate gene essentiality.
• Based on ClosTron targeted knockout following engineered gene duplication.
• Gene duplication rapidly achieved at the *pyrE* locus using Allele-Coupled Exchange.
• *trpS*, *metK,* and *CD0274* shown to be essential in *Clostridium difficile* 630Δ*erm*.
• *CD0274* (*dhaT*) could represent a potential drug target.

*Clostridium* is a bacterial genus composed of around 100 species which are either of industrial or medical importance. Arguably, the most noteworthy clostridial species are those that cause human and animal diseases, and in particular *Clostridium difficile*. Responsible for *Clostridium difficile*-associated disease (CDAD), it is the leading cause of hospital-acquired and antibiotic-associated diarrhoea worldwide. In the US, *C. difficile* was responsible for almost half a million infections and 29,000 deaths in 2011, while similar rates of infection are estimated in Europe [1], [2]. Treatment options remain limited [3]. Moreover, the emergence of strains resistant to currently used antibiotics [4] has led the CDC to include *C. difficile* as one of the major “Antibiotic Resistance Threats in the United States, 2013” [5]. New therapies are required.

One option is to identify those genes and their products which are essential for the bacterium's survival, and then develop interventions that target that function. Direct demonstration of gene essentiality is not, however, a straightforward process. One indirect approach, is to use high-throughput sequencing methods (Transposon-Directed Insertion Site Sequencing, TraDIS) to allow simultaneous screening of saturating transposon libraries. Those genes found not to contain transposon insertions are presumed to be essential for growth under the conditions used to make the library [6]. When applied to the *C. difficile* strain R20291, 404 genes were suggested to be essential [7]. Another strategy, and that adopted here, is to show that a particular gene can only be inactivated if the target cell is made a merodiploid through the addition of a second functional copy of the gene in question [8]. Our approach was made possible by the properties and characteristics of two clostridial gene tools: (i) the ClosTron [9], a Group II intron retargeting mutagen which is absolutely reliant on the presence of a specific recognition sequence within the gene to be inactivated, and (ii) Allele-Coupled Exchange (ACE) technology, which allows the rapid integration of DNA of any size or complexity into the *C. difficile* genome [10].

**Fig. 1:**
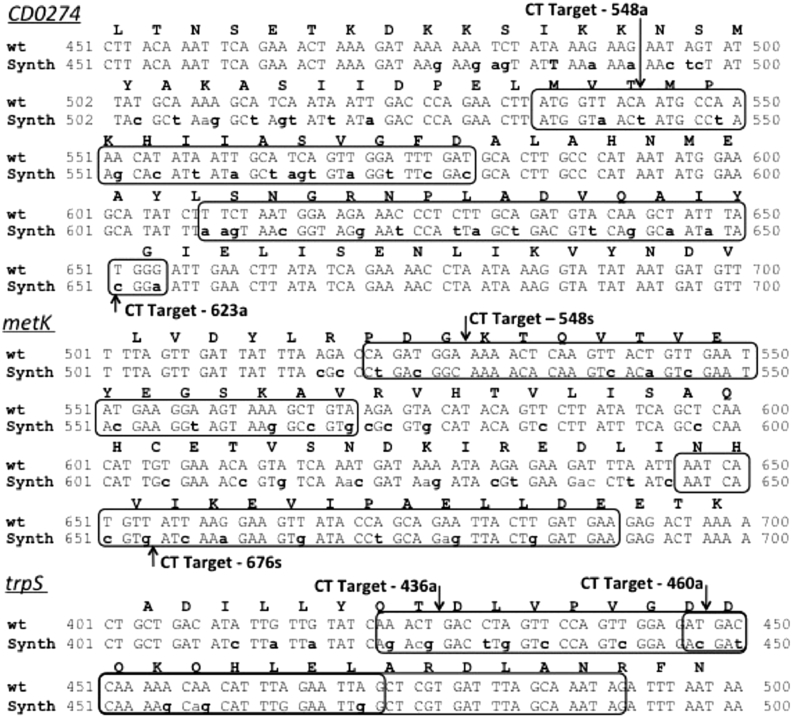
Protection of the synthetic merodiploid copy of the candidate essential gene. The ClosTron group II intron recognises a 45mer target site on the sense (s) or anti-sense strand (a) displayed by a box encompassing the two target sites for each candidate essential gene. The native gene (wt) is shown above, and the re-synthesised gene below (Synth). The degenerate changes made to synthetic gene codons are shown in bold lowercase. Those changes made outside of the boxed target regions represent alterative ClosTron target sites not used in the study.

For proof of principle studies we chose *metK* (S-adenosylmethionine synthetase) and *trpS* (tryptophan tRNA synthetase) as both have been shown to be essential in *Bacillus subtilis* [11] and were included in the list of 404 essential *C. difficile* genes identified using TraDIS [7]. Retargeted ClosTrons directed against each gene were designed and constructed using standard procedures [9]. In parallel, both genes were resynthesized exactly as the wildtype, except over the two 45 nucleotide regions that encompassed two predicted ClosTron target sequences. Here, several synonymous codon replacements were made, in order to change the DNA sequence without affecting the amino acid sequence of the encoded MetK or TrpS protein (Fig. 1). As a consequence, these regions were no longer recognised as intron targets using the Peruka algorithm [12]. In each case, care was taken not introduce any rare-codons into the amino acid sequence so the protein's translational efficiency would be as close as possible to the native proteins.

**Fig. 2:**
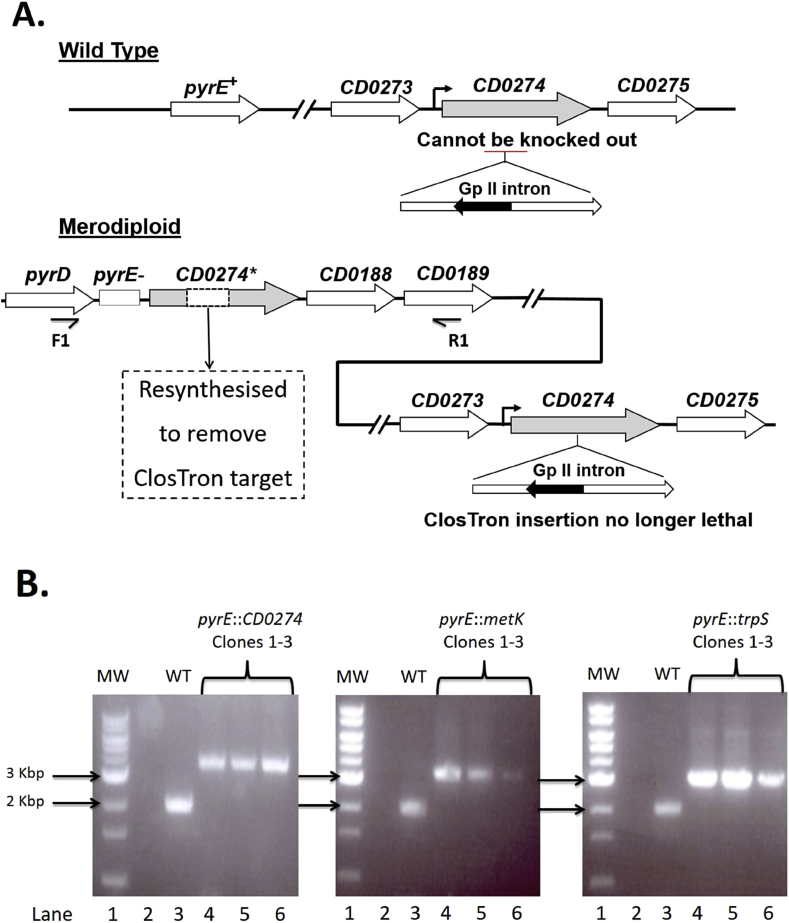
Construction of target gene merodiploid cell lines. (**A**) The synthetic gene (*CD0274*) lacking the intron target (dashed box) to be delivered is cloned between the two homology arms of the replication defective vector, pMTL-JH18, and integrated into the genome using ACE [10]. Concomitant with integration, the *pyrE* gene is inactivated resulted in a cell that is auxotrophic for uracil and resistance (^R^) to fluoroorotic acid (FOA). Insertion of the group II intron is selected on the basis of acquisition of resistance to erythromycin due to acquisition of the activated *ermB* gene (indicated as a filled arrow). Insertion of the intron into the original CD0274 gene only takes place in the merodiploid cell and not the wild type strain. The position of the two PCR primers F1 (Cdi630:pyrD-F1) and R1 (Cdi630:CD0189-R1) used to confirm insertion of the merodiploid gene at the *pyrE* locus is shown beneath the relevant region of the genome. (**B**) PCR screening of three double crossover clones of each target gene (*CD0274*, *metK* and *trpS*) using primers Cdi630:pyrD-F1 (F1) and Cdi630:CD0189-R1 (R1). The molecular weight marker (MW) used (lane 1) is an NEB 1 Kbp DNA Ladder. Lane 2 on all three gels is water only negative control, lane 3 is the wildtype (expected size ∼2 kbp) *C. difficile* 630Δ*erm* DNA control, lanes 4 are candidate clones of the three target genes as indicated (expected size ∼ 3.3 kbp).

The ACE integration vector pMTL-JH18 [10] is designed (through provision of flanking asymmetric homology arms) to create a deletion in the *pyrE* gene (encodes orotate phosphoribosyl-transferase) which confers on the host resistance to 5-fluorouracil (FOA). The two synthetic genes including their natural promoter, were cloned into pMTL-JH18 [10], transformed into *C. difficile* 630Δ*erm* and plated on media supplemented with thiamphenicol. Single crossover integrants, selected on the basis of their larger colony size, were plated onto minimal media containing FOA and uracil [10]. The majority of the FOA-resistant (^R^) colonies that arose (e.g. 16 of 17 in the case of *metK*) were clones in which the *metK or trpS* had integrated into the genome concomitant with inactivation of the *pyrE* locus. Authenticity was confirmed by undertaking a PCR screen employing two primers flanking *pyrE* (Fig. 2). Those few FOA^R^ colonies which arose that lacked an insertion most likely represented spontaneous mutants in *pyrE* or *pyrF.*

The resulting merodiploids for *metK* and *trpS* were respectively used in parallel with the wild type 630**Δ***erm* strain, as recipients in ClosTron mutagenesis experiments directed against the native *metK* and *trpS* genes. Following the transfer of the four retargeted plasmids (two targets for each gene) to both the wild type and the appropriate merodiploid strain, putative ClosTron mutants were isolated as erythromycin-resistant (Em^R^) colonies. In the case of the wild type 630**Δ***erm* strain, despite screening 70 such Em^R^ clones for each target, no intron insertions in either *metK* or *trpS* were detected. In contrast, intron insertions were readily found when a merodiploid strain was the recipient, with the majority of the 20 Em^R^ colonies tested for each target being intron insertions in either *metK* or *trpS* (Table 1). These data demonstrate that the *metK* and *trpS* genes are essential under the growth conditions employed and cannot be inactivated unless a second, functional copy of the gene is also present in the cell.

Having demonstrated the utility of the system, *CD0274*, a previously [13] suspected essential gene identified and annotated as *gldA* (glycerol dehydrogenase) in *Clostridium beijerinckii* and *dhaT* (1,3-propandiol oxidoreductase) in *C. difficile,* was used to validate the method. *CD0274* is proposed to play a pivotal role in the detoxification of the toxic metabolite methylglyoxal (MG) [13], [14]. Accordingly, the CD0274 gene was synthesised as before and the sequences of the two highest scoring [12] ClosTron targets altered through the use of degenerate codon sequences (Fig. 1). The synthetic gene was sub-cloned into pMTL-JH18 [10] and integrated into the genome at the *pyrE* locus using ACE [10]. Following the constructing of the *CD0274* merodiploid, we were able to show that ClosTron mutants could be obtained with high efficiency in this strain, but not in the parent 630Δ*erm* wild type strain (Table 1). Previously, mutants made in this gene using single crossover plasmid integration developed no further than pin-prick colonies, and could not be propagated further [13]. Our data re-inforces the view that this gene is essential and could represent a potential drug target for combating *C. difficile* infections. Interestingly, the equivalent gene in R20291 (CDR20291_*0278* but misannotated as *metE*) was not designated as essential in the TraDIS study [7].

In this study, the necessary merodiploid cell was created through ACE-mediated inactivation of the *pyrE* locus. An alternative option would be to use a *pyrE* minus host and introduce the second copy of the gene into the genome concomitant with correction of the *pyrE* allele to wildtype. Restoration of uracil prototrophy (growth on minimal media without uracil supplementation) represents a simpler selective phenotype. More importantly, because the *pyrE* deletion mutation cannot revert, there can be no false positives. The required *pyrE* minus mutants of *C. difficile* strains R20291 and 630 are available [15], and are relatively easily created in any *Clostridium* [16].

In conclusion, we have developed a simple and rapid method that may be used to experimentally confirm essentiality in, but not limited to, *C. difficile*. Using this system, we demonstrated that *trpS*, *metK,* and *CD0274* are essential in *C. difficile* 630Δ*erm*. Our method may be used to confirm suggestions of essentiality that arise from genome-wide mutational methods such as TraDIS.

## Authorship/contribution

DJFW undertook all the experimental work described in the study, with guidance from JTH, KW and NPM. JTH and NPM conceived the study. NPM and DJFW wrote the manuscript. All authors read and commented on the manuscript.

## Figures and Tables

**Table 1:** ClosTron Group II intron Insertion Frequencies at Target Gene.

Target gene	ClosTron target	Wild type recipient			Merodiploid recipient		
		Colonies screened	Intron insertions	Frequency (%)	Colonies screened	Intron insertions	Frequency (%)
*metK*	548s	70	0	0	20	20	100
	676a	70	0	0	20	20	100
*trpS*	548s	70	0	0	20	20	100
	623a	70	0	0	20	18	90
*CD0274*	436a	70	0	0	20	15	75
	460a	70	0	0	20	20	100
