# Supplementary material for: A genetic assay for gene essentiality in Clostridium
Source: Anaerobe. 2016 Dec;42:40–3. doi: 10.1016/j.anaerobe.2016.07.007 (PMC5154367; doi:10.1016/j.anaerobe.2016.07.007)
Supplement: Supplementary file 1 [file mmc1.docx]

**SUPPLEMENTARY INFORMATION**

**A genetic assay for gene essentiality in *Clostridium***

David J F Walker^1 ª^, Klaus Winzer^1^, John T Heap^1 b^, and Nigel P Minton^1,2*^

^1^ *Clostridia Research Group, BBSRC/EPSRC Synthetic Biology Research Centre (SBRC), , School of Life Sciences, Centre for Biomolecular Sciences , University of Nottingham, University Park, Nottingham, NG7 2RD, United Kingdom*

^2^ *Nottingham Digestive Disease Centre, NIHR Biomedical Research Unit, The University Of Nottingham, University Park, Nottingham, United Kingdom.*

**Materials and Methods**

#### *Bacterial strains, growth conditions, and plasmid transfer*

#### *Escherichia coli* TOP10 (Invitrogen) and *E. coli* CA434 [Purdy *et al.,* 2002] were grown in Luria-Bertani (LB) media supplemented with chloamphenicol (25 µg/ml) where appropriate. *C. difficile* strains were grown in BHIS agar or broth supplemented with *C. difficile* supplement (Oxoid), thiamphenicol (15 µg/ml), or erythromycin (2.5 µg/ml or 10 µg/ml), where appropriate. The *C. difficile* single-crossover ACE mutants were grown on *C. difficile* minimal media [Cartman and Minton, 2010] supplemented with 5-Fluoroorotic Acid (5-FOA) (1 mg/ml) and uracil (5 µg/ml). Double-crossover ACE mutants displaying the *pyrE*^-^ genotype were grown on BHIS supplemented with 5 µg/mL uracil. All media used for growth of *C. difficile* was pre-reduced by overnight incubation in an anaerobic environment. Unless specified otherwise, all *E. coli* and *C. difficile* strains were incubated at 37 °C. *E. coli* TOP10 was used as a cloning strain for construction of the different plasmids. Plasmids were transferred into *C. difficile* via the *E. coli* donor strain CA434 by conjugation as previously described [Purdy *et al*., 2002].

#### *Construction of the retargeted intron vector for gene inactivation*

#### Target sites in the three candidate essential genes were identified using the Perutka algorithm [Perutka *et al.,* 2004]. DNA was ordered from DNA2.0 and the appropriate pMTL007C-E2 plasmids were built as previously described [Heap *et al.,* 2010]. Each ClosTron plasmid containing the targeted intron is named according to nomenclature described by Heap *et al*., [2010].

**Table S1.**

List of primers used in this study.

| **Primer** | **Primer Sequence 5’ to 3’** |
| --- | --- |
| Cdi630:dhaT-F1 | GAAATAGGAGGTTTTAGAATGAATTTTAATTATAATTTGCC |
| Cdi630:dhaT-R1 | TAAATATGTAAAGTCAAATTATAAAGACTTATGGTACATTTCC |
| Cdi630:PdhaT-F1 | ATATCGCGGCCGCTCTAATAAAAAACTCTATTTAGTTAAATATAGTTAACTTG |
| Cdi630:PdhaT-R1 | AGTAACCATATGAAAACCTCCTATTTCTACTTTAGTAATAATTTTATGTTTTTAACAAAGTG |
| Cdi630:metK-F1 | CAATAGGAACACCCCCTTCA |
| Cdi630:metK-R1 | GCAACATAAGCTCCAGCAAA |
| Cdi630:trpS-F1 | CCCCTTTGAGATGAGCAAAA |
| Cdi630:trpS-R1 | TAAGGCAGATGGAAGTGCTG |
| Cdi630:pyrD-F1 | GATGGGCGGAATAACTAAAGC |
| Cdi630:CD0189-R1 | GGTTCTGGAACCAGAGATTATTTAG |
| Cdi630:dhaT-inF1 | TGCAGAAGATTGTTCAGATGC |
| Cdi630:trpS-inF1 | AAAGCAGCATTTGGAATTGG |
| Cdi630:trpS-inR1 | GATGGCTGTGCACCACTAAA |
| Cdi630:metK-inR1 | GCAACATAAGCTCCAGCAAA |
| EBS Universal | CGAAATTAGAAACTTGCGTTCAGTAAAC |

####

#### *Construction of the CD0274 merodiploid plasmid*

#### All primers used in this study are listed in Table S1. The target gene *CD0274* was PCR amplified using primers Cdi630:dhaT-F1 and Cdi630:dhaT-R1 which introduce restriction sites *Nde*I and *Nhe*I plus His-tag at the 5’ and 3’ of *CD0274*, respectively. The PCR product was then cloned into pMTL82151 using the restriction endonucleases *Nde*I and *Nhe*I. The 289-bp segment of DNA between the two *Sca*I restriction endonuclease sites within *dhaT* was redesigned and synthesised by DNA2.0 to change the nucleotide sequence, but keep the amino acid sequence the same. The synthetic fragment was cloned into pMTL82151-dhaT using the restriction endonuclease *Sca*I to replace the native segment, giving rise to vector pMTL82151-dhaT-S. The native promoter of *dhaT* was PCR amplified with primers Cdi630:PdhaT-F1 and Cdi630:PdhaT-R1 and cloned into vector pMTL82151-dhaT-S, giving rise to pMTL82151-PdhaT-dhaT-S.

#### *Construction of the allelic-coupled exchange (ACE) vector to create a merodiploid*

#### Both dhaT-S and its native promoter were cloned from pMTL82151-PdhaT-dhaT-S into the ACE vector pMTL-JH18 [Heap *et al.,* 2012] with restriction endonucleases *Not*I and *Nhe*I, creating vector pMTL-JH18-dhaT. Construction of *metK* and its native promoter was carried out by DNA2.0 by synthesising the section with changes in the nucleotide sequence between nucleotides 516 and 686 but keeping the same amino acid sequence as the native *metK*. During synthesis, restriction endonuclease sites *Not*I and *Nde*I were introduced at the 5’ and 3’ ends of the segment, respectively. The synthetic *metK*-S and its promoter were then cloned from DNA2.0’s in-house vector into pMTL-JH18 using the restriction endonucleases *Not*I and *Nhe*I, creating vector pMTL-JH18-metK. In the case of *trpS*, the *Clostridium sporogenes* thiolase promoter was substituted for the native promoter which drives *trpS* transcription. DNA2.0 synthesised *trpS* with changes in the nucleotide sequence between nucleotides 407 and 441, the thiolase promoter upstream of *trpS*-S, and introduced restriction endonuclease sites *Not*I and *Nhe*I at the 5’ and 3’ end of the segment respectively. The synthetic *trpS-S* and synthetic thiolase promoter was then cloned into pMTL-JH18 using the restriction endonucleases *Not*I and *Nhe*I, creating pMTL-JH18-trpS.

#### *Gene targeting in C. difficile*

#### The six different pMTL007C-E2 re-targeted ClosTron plasmids were transferred into *C. difficile* 630∆*erm* by conjugation from the *E. coli* donor strain CA434 as previously described [Purdy *et al.,* 2002]. Thiamphenicol-resistant transconjugants which appeared between 48-72 h after inoculation were re-streaked onto fresh plates to ensure purity. Single colonies containing the appropriate plasmids were then re-suspended in 100 µl PBS and plated onto BHIS plates supplemented with *C. difficile* supplement and Erythromycin (2.5 µg/ml) to select for integration of the group II intron into the target and gene and subsequent activation of the erythromycin RAM. Independent erythromycin-resistant colonies were selected for further analysis by replica-plating on erythromycin (2.5 µg/ml) and thiamphenicol (15 µg/ml) to test for pMTL007C-E2 plasmid loss. Chromosomal DNA was isolated using the Qiagen DNeasy kit following the manufacturer’s instructions. PCR screening to identify correct insertion of the intron into the target gene was carried out on the purified genomic DNA. In the instance of *CD0274*, the primers Cdi630:dhaT-R1 and the EBS Universal were used to amplify across the target gene/intron junction of *CD0274*, the second PCR using *CD0274* flanking primers Cdi630:dhaT-F1 and Cdi630:dhaT-R1 were used to amplify across *CD0274* and the integrated intron. The same screening technique was carried out for the other ClosTron targets targeted against; *metK* using the primers Cdi630:metK-F1 and EBS universal for the gene/intron junction and primers Cdi630:metK-F1 and Cdi630:metK-R1 for flanking the gene and insertion site, and for *trpS* using the primers Cdi630:trpS-R1 and the EBS universal for the gene/intron junction and the primers Cdi630:trpS-F1 and Cdi630:trpS-R1. Confirmation of the correct insertion was carried out by sequencing the PCR product of the gene/intron junction.

#### *Isolation and PCR screening of essential gene merodiploids*

#### The three synthetic merodiploid variants of pMTL-JH18 containing the protected copy of the candidate essential gene were conjugated into *C. difficile* 630∆*erm*. Thiamphenicol-resistant transconjugants were re-streaked onto fresh media to ensure purity. Due to the nature of the pseudo-suicide replicon present on the pMTL-JH18 backbone, the larger colonies present on the plate, suggesting single-crossover has occurred, were selected and re-streaked onto fresh media. This was repeated until single-crossover mutants were obtained and confirmed by PCR using primers Cdi630:pyrD-F1 and Cdi630:dhaT-inF1 in the case of pMTL-JH18-dhaT, and Cdi630:metK-inR1 and Cdi630:trpS-inR1 for pMTL-JH18-metK and pMTL-JH18-trpS, respectively. Counter-selection for the second-crossover event was carried out by plating the single-crossover mutants onto *C. difficile* minimal media supplemented with 5-FOA (1 mg/ml) and uracil (50 µg/ml). Colonies resistant to 5-FOA were selected after 24 h and plated onto fresh media. Colonies which grew after 24 h were re-streaked onto BHIS agar supplemented with uracil (50 µg/ml) and PCR screened. PCR screening for the double-crossover event to ensure insertion of each of the protected essential genes and removal of the of the plasmid, primers Cdi630:pyrD-F1 and Cdi630:CD0189-R1 were used to amplify across the insertion site. Confirmation of correct insertion was carried out by sequencing the PCR product of each of the PCR reactions.

#### References

Cartman ST, Minton NP (2010) A mariner-based transposon system for in vivo random mutagenesis of *Clostridium difficile*. Appl Environ Microbiol 76(4):1103-1109. doi:10.1128/AEM.02525-09

Heap JT, Kuehne SA, Ehsaan M, Cartman ST, Cooksley CM, Scott JC, Minton NP. The ClosTron: Mutagenesis in *Clostridium* refined and streamlined. J Microbiol Methods 2010;80:49-55. doi: 10.1016/j.mimet.2009.10.018.

Heap JT, Ehsaan M, Cooksley CM, Ng YK, Cartman ST, Winzer K, Minton NP. Integration of DNA into bacterial chromosomes from plasmids without a counter-selection marker. Nucleic Acids Res 2012;40(8):e59. doi:10.1093/nar/gkr1321.

Heap JT, Pennington OF, Cartman ST, Carter GP, Minton NP. The ClosTron: A Universal gene knock-out systen for the genus *Clostridium*. J Microbiol Methods 2007;70:452-464. doi:10.1016/j.mimet.2007.05.021.

Perutka J, Wang W, Goerlitz D, Lambowitz AM. Use of computer-designed group II introns to disrupt *Escherichia coli* DExH/D-box protein and DNA helicase genes. J Mol Biol. 2004: J Mol Biol 2004;336(2):421-39. doi:10.1016/j.jmb.2003.12.009

Purdy D, O'Keeffe TA, Elmore M, Herbert M, McLeod A, Bokori-Brown M, et al. Conjugative transfer of clostridial shuttle vectors from *Escherichia coli* to *Clostridium difficile* through circumvention of the restriction barrier. Mol Microbiol 2002;46(2):439-452. DOI: 10.1046/j.1365-2958.2002.03134.x
